# Head Lice Infestation: An Unusual Cause of Iron Deficiency Anemia in a 13-Year-Old Female

**DOI:** 10.7759/cureus.25956

**Published:** 2022-06-15

**Authors:** Chukwunonye O Ogbuji, Alexis Schuck, Matthew DeVries, Elleana J Majdinasab, Kevin Benson, Samer Zaid-Kaylani, Smita Bhaskaran

**Affiliations:** 1 Pediatric Medicine, Texas Tech University Health Sciences Center, Amarillo, USA; 2 School of Medicine, Texas Tech University Health Sciences Center, Amarillo, USA

**Keywords:** blood loss, iron deficiency anemia, pediculosis, pediculus humanus capitis, head lice infestation

## Abstract

Pediculosis is a parasitic infestation of the human head and body by *Pediculus humanus. *This is a benign condition commonly seen in children and capable of causing severe pruritus. The parasite thrives on human blood and in some cases, the volume of blood loss over time could be large enough to precipitate anemic symptoms in the patient. We describe the case of a 13-year-old girl who presented with shortness of breath on exertion, palpitations, and easy fatigability. An incidental finding of *Pediculus humanus capitis* infestation was made during physical examination. Complete laboratory investigations did not reveal other possible causes of anemia, leading to a diagnosis of iron deficiency anemia (IDA) secondary to severe chronic pediculosis. This case highlights a rare and unusual cause of IDA in children caused by pediculosis, and the need for a thorough investigation, close follow-up, and treatment.

## Introduction

Pediculosis capitis (head lice) among school-aged children is a matter of public health concern. In the United States, about 6-12 million infestations occur each year in children aged three to 12. It has a benign course and patients make full recovery with appropriate treatment [1]. Lice are obligate ectoparasites that feed on human blood, and in rare cases, the parasite burden can be high enough to cause iron deficiency anemia (IDA). Blood loss with severe infections is estimated to be between 0.008 ml/day to 0.7 ml/day [2]. Some cases of severe pediculosis capitis infestation leading to iron deficiency anemia have been reported both within and outside of the United States [2].

Background

Iron deficiency is the leading cause of anemia in the United States [3]. It can stem from poor acquisition of iron or the loss thereof through bleeding. Women are more frequently affected by IDA than men, with a lifetime incidence of 11% and 1%, respectively [4]. The risk is higher in women due to menstrual blood loss and pregnancy. It has been estimated that the average amount of blood loss in a menstrual cycle for a mature post-pubertal female is 34 mL and women who lost between 40 and 60 mL of blood during menses were frequently found to be anemic [5]. Pregnancy in women causes iron deficiency because fetal growth and development require increased iron demands, and the maternal blood supply must expand to meet those demands. About 37% of pregnant women in developed countries experience iron deficiency during the first trimester [6].

Another major source of blood loss in the general population is gastrointestinal (GI) pathology. Though many of these are more common in adults, some may also impact children. This occurs by the gradual loss of small volumes of blood over time. GI cancers (colon, esophageal, gastric, and small intestinal), although not frequently seen in the pediatric population, are common causes of GI blood loss leading to IDA. Other GI pathologies implicated are Meckel’s diverticulum, celiac disease, vascular malformations, inflammatory bowel diseases, and parasitic infestation (hookworms) [7]. Hookworms are an example of the profound impact that chronic infestation with a hematophagous parasite can make. Though these parasites only draw a small amount of blood, over time the cumulative effect can be significant. Outside the GI system, pediculosis represents a similar, albeit rare, case of excessive burden and prolonged exposure causing meaningful blood loss [7].

Pediculosis is an infestation with the human head and body louse, *Pediculosis humanus*, and is thought to be another potential cause of iron-deficiency anemia in severe cases. Pediculosis is caused by two known subspecies of the Anoplura insect, *Pediculosis humanus capitis* (head lice) and *Pediculosis humanus corporis* (body lice). Children aged three to 12 years are mostly affected, with females having a higher reported prevalence than males [8]. Head lice infestation crosses socioeconomic boundaries, while body lice primarily affect the homeless population and people with limited access to hygienic resources [7]. *Pediculus humanus capitis* is an obligate ectoparasite known to feed only on human blood, thus possibly causing hematologic issues in the host. Pruritus is the most common symptom of this condition, occurring in approximately 14% to 36% of cases; however, most cases are asymptomatic [9]. The itching is presumed to be caused by an allergic reaction to the insect’s saliva. The clinical course of *Pediculus humanus capitis* is generally well-tolerated and benign if managed properly, but inadequate treatment may lead to excessive parasite burden and anemia from chronic blood loss [10].

## Case presentation

**Figure 1:**
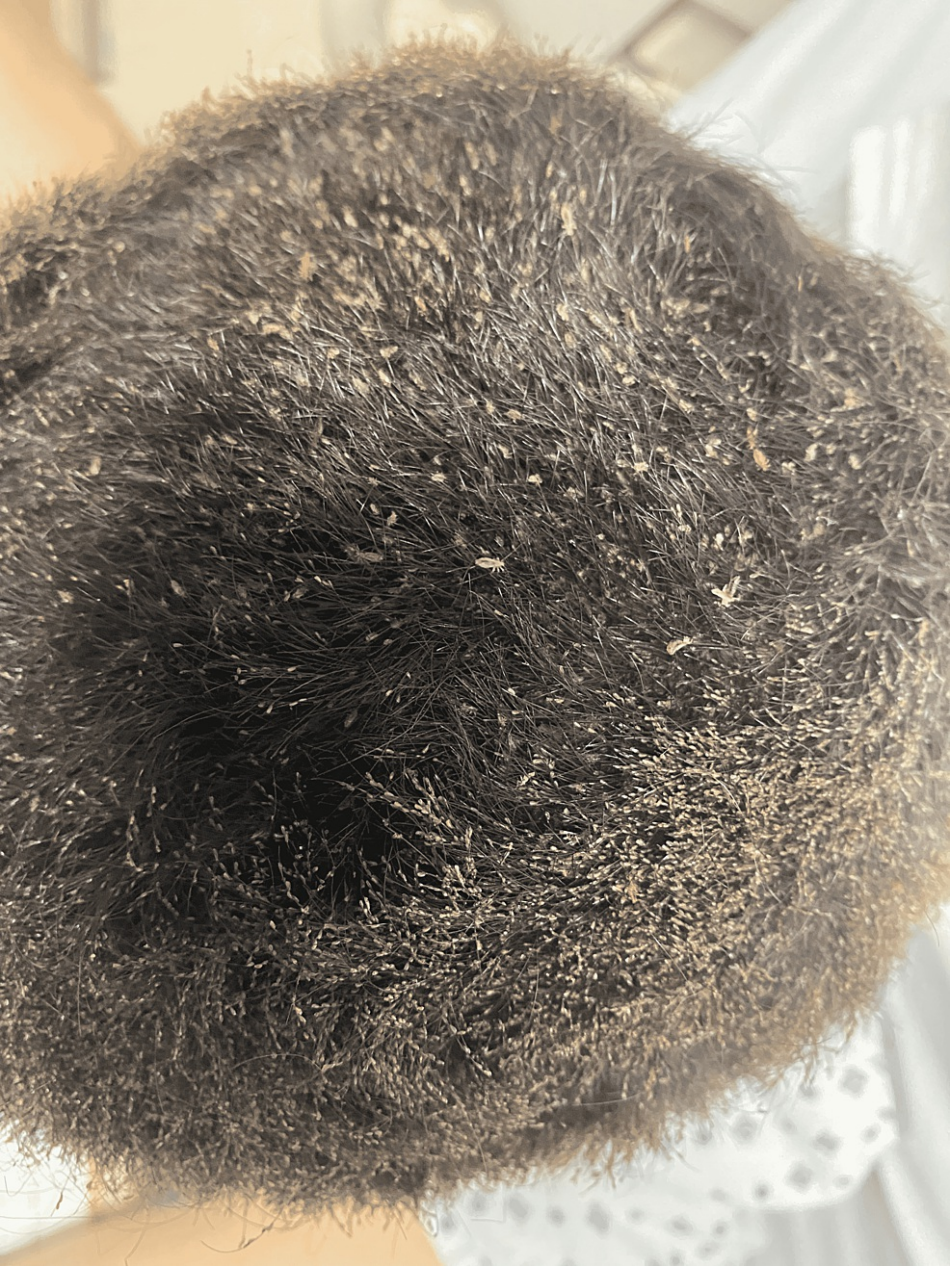
Patient's hair after being shaved showing visible parasites

We present the case of a 13-year-old female admitted through the outpatient clinic for complaints of tiredness, dizziness, and palpitations for about a month duration. Prior to her presentation, she was unable to climb a flight of stairs without a break or assistance. Nutritional history revealed balanced meals with adequate consumption of iron-rich food and no history of excessive cow milk intake. She was two years post-menarche and had regular cycles with no overflows or any history suggestive of menorrhagia. She denied passage of dark tarry stools, bleeding from any sites, or recent loss of consciousness. Vital signs on admission showed a temperature of 99.5F; blood pressure of 118/70 mmHg; respiratory rate of 20cpm; heart rate of 121bpm and oxygen saturation of 99% on room air. She had a height of 156.5cm, a weight of 61.6kg, and a BMI of 25.15kg/m2. Physical examination revealed severe conjunctival pallor, no audible murmurs, and an incidental finding of head lice infestation. The lice burden was heavy with visible nits (Figure 1) and excoriations all over the body due to excessive itching.

Initial laboratory values showed a hemoglobin of 5.3g/dL, with a mean corpuscular volume (MCV) of 71 and a reticulocyte count of 2%. WBC was 5.3 x 10^3^ with significant eosinophilia (14.7%), and platelet count was 389,000. Iron studies showed a total iron-binding capacity (TIBC) of 521, a ferritin level of 2.6ng/mL, and a serum iron level of 464 ug/dL. She had taken a tablet of iron prescribed at the outpatient clinic some hours prior to initial blood samples being drawn. Haptoglobin level and lactate dehydrogenase were 111mg/dL and 273units/L respectively. A complete metabolic panel showed a creatinine level of 0.6mg/dL, indicative of adequate renal function and erythropoiesis.

Parents gave a history of living in an old house with refurbished paints; however, the patient’s blood lead level was less than 1mcg/dL. Hemoglobin electrophoresis demonstrated normal hemoglobin with no variants or thalassemia. Peripheral blood smear reported cells to be microcytic and hypochromic with moderate anisocytosis and poikilocytosis. There were no teardrop forms, target cells, or schistocytes. The stool was negative for *Giardia* and *Cryptosporidium* antigens as well as *Helicobacter pylori* antigen. An evaluation for rheumatologic disorders was pan-negative for antinuclear antibodies (ANA) comprehensive panel. A thorough social and sexual history, alongside genital examination, was conducted on the patient to rule out possibilities of sexual abuse and co-infestation with *Pediculosis pubis*, a sexually transmitted crab louse (*Phthirus pubis*) infestation. She had no history of sexual contact, and her genital area was devoid of lice infestation.

The patient had several previous failed treatments for head lice prior to our encounter. She was admitted and advised to have her hair trimmed down. This eradicated a large heap of parasites but the burden remained significantly heavy. She was treated with topical permethrin in addition to oral ivermectin due to previous treatment failures. She also received 3 units of blood over the course of her three-day admission. On the day of discharge, her hemoglobin was 9.8g/dL with an MCV of 80, her pallor and palpitations had resolved, and she was more effort tolerant. During outpatient follow-up five days post-discharge, she had hemoglobin and hematocrit levels of 12g/dL and 38% respectively, with an MCV of 86.6fL.

## Discussion

Although menorrhagia is one of the most common causes of IDA in young females, other notable risk factors can contribute as well. Such factors include increased iron requirements among adolescents aged 13-17 years, pregnant individuals, overweight adolescents, and young athletes. Furthermore, a decrease in iron absorption from the gut, seen in conditions such as celiac disease, inflammatory bowel diseases, autoimmune atrophic gastritis, and *H. pylori* infection, can also lead to iron deficiency anemia [11]. Herein we have presented another potential etiology for IDA. While this is worth considering, these alternative underlying causes should be considered and ruled out prior to making a diagnosis of IDA secondary to chronic pediculosis.

A causal relationship between pediculosis capitis and IDA has not been established, but cases have been reported both within and outside the United States. Guss et al. conducted a case series regarding lice infestation among a homeless population. The findings suggested that study subjects shared common factors such as lack of access to basic amenities like safe housing and adequate hygiene products [12]. Similarly, the 13-year-old patient in this case report also had notably decreased personal hygiene on admission (although she was not experiencing homelessness). Her social history was significant for a lack of running water at home, which created a barrier to regular baths and maintenance of healthy levels of personal hygiene.

Some pediatric and adult cases have been reported in the literature attributing IDA to an underlying lice infestation. Ronsley et al. reported a four-year-old female with a hemoglobin of 2.1g/dL [13]; Hau and Muhi-Iddin reported an 11-year-old male who presented with a hemoglobin of 4.2g/dL [14] while Fustino et al. also reported the case of a 12-year-old female who presented with a hemoglobin 4.7g/dL [15]. All three subjects had severe head lice infestations. A case by Althomali et al. showed a 23-year-old female with chronic lice infestation presenting with a hemoglobin level of 2.2g/dL [16]. A 32-year-old male and a 74-year-old female, both with schizophrenia, have also been diagnosed with IDA secondary to lice infestation in cases reported by Batool et al. and Woodruff et al. respectively [17,18]. In all these cases, other causes of iron deficiency were ruled out. Some of these patients had comorbidities that may have affected their ability to obtain enough iron through their diet, including advanced age and psychiatric disorders.

## Conclusions

This case highlights the importance of having a holistic approach to the management of anemia in pediatric patients. Severe IDA secondary to pediculosis can be a diagnosis of exclusion. Having ruled out possible nutritional causes of anemia, hematological disorders such as thalassemia and sickle cell disease, losses through the gastrointestinal tract, and menstruation, we were left with blood loss secondary to lice infestation as the only potential cause of anemia in this child.
